# Superior Mesenteric Artery Syndrome: A Case Report

**DOI:** 10.7759/cureus.91747

**Published:** 2025-09-06

**Authors:** Joseph Shen, Kevin Shen, Brandon Weissman, Shafayath Chowdhury, Roy Shen

**Affiliations:** 1 Dermatology, Lake Erie College of Osteopathic Medicine, Greensburg, USA; 2 Medical School, Lake Erie College of Osteopathic Medicine, Greensburg, USA; 3 Otolaryngology, Lake Erie College of Osteopathic Medicine, Elmira, USA; 4 Anesthesiology and Critical Care, Lake Erie College of Osteopathic Medicine, Elmira, USA; 5 General Surgery, Tufts University School of Medicine, Boston, USA

**Keywords:** esophagogastroduodenoscopy, general surgery, sma syndrome, superior mesenteric artery (sma), surgery

## Abstract

Superior mesenteric artery (SMA) syndrome is a rare condition characterized by the narrowing of the space between the SMA and the aorta, resulting in the compression of the third portion of the duodenum. This syndrome has many names, including cast syndrome, arterio-mesenteric duodenal compression syndrome, and Wilkie syndrome. This is attributed to the loss of the intervening mesenteric fat pad, posing diagnostic and therapeutic challenges due to its nonspecific presentation. We present a case of a male in his 50s, who arrived at the emergency room with complaints of episodes of nausea, vomiting, and abdominal pain with unremarkable laboratory studies, other than being hypokalemic and an acute elevation of white blood cell count. After the patient was stabilized, a computed tomography (CT) scan revealed compression at the junction of the second and third portions of the duodenum, which was later confirmed by an esophagogastroduodenoscopy (EGD) evaluation. Initially, a jejunostomy tube (J-tube) was placed for feeding purposes.

## Introduction

Superior mesenteric artery (SMA) syndrome was first described in the 1800s as a cause of proximal small bowel obstruction [1]. SMA syndrome’s incidence is estimated to be 0.1-0.3%. It most often affects individuals aged 10 to 39 years [1]. This syndrome manifests from compression of the third portion of the duodenum between the SMA and the aorta. This compression is due to loss of the mesenteric fat pad that rests between the aorta and the SMA [2]. This condition is characterized by an aortomesenteric angle of less than 25 degrees, which creates a clamping effect on the duodenum [1]. For reference, the normal aortomesenteric angle ranges from 38 to 65 degrees [3]. Any condition associated with weight loss may precipitate this syndrome, as it disrupts gastrointestinal absorption and exacerbates symptoms such as vomiting, ultimately resulting in a vicious cycle. When SMA syndrome is suspected, imaging is often recommended. Imaging options include plain film X-ray, endoscopy, computed tomography (CT), ultrasound, and magnetic resonance angiography (MRA) [1]. Oftentimes, patients also experience electrolyte disturbances such as hypokalemia, hypochloremia, and metabolic alkalosis [1]. Treatment initially involves medical management, including stabilization of any possible electrolyte abnormalities and restoration of normal fluid levels. A nasogastric tube can also be placed for decompression [1]. Nutritional support is the pillar for conservative therapy to increase the fat pad between the aorta and the SMA. Patients who do not respond to medical treatment often require surgical intervention with a wide array of endoscopic options [1].

## Case presentation

A male in his 50s with a history of quadriplegia secondary to a C4 fracture, with complications including orthostatic hypotension, neurogenic bladder, and a chronic suprapubic Foley catheter placement, presents at the ER with a one-day history of nausea, vomiting, and upper abdominal pain. His last oral intake was noted to be one day ago, accompanied by intermittent fevers.

Notably, he presented with a similar set of symptoms three weeks prior, was diagnosed with colitis, and treated with ceftriaxone and metronidazole. Since that time, he has experienced no subsequent changes in his bowel movements and continues to follow a bowel regimen. His surgical history is significant for a gastric perforation repair; the exact technique used is unknown. Upon examination, he has sinus tachycardia with a heart rate of 114 beats per minute and hypokalemia, with a potassium level of 3.3 millimoles per liter (mmol/L), and a mild elevation in white blood cell count at 11.3 × 10^9^/10^9^/liters (L). He also presented lactate levels of 1.8 mmol/L and glucose levels of 128 mmol/L. Although the values were out of range, it was difficult to determine the patient’s condition due to the possibility that it was a natural stress-induced response.

**Figure 1:**
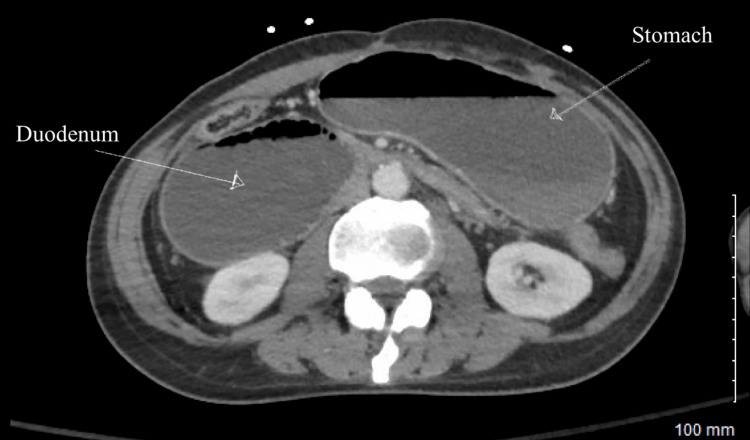
Axial computed tomography scan displaying the compressed duodenum and the dilated stomach In this image, the compressed duodenum and dilated stomach due to obstruction in gastric emptying were captured. This suggests that the patient exhibits characteristics associated with superior mesenteric artery (SMA) syndrome.

**Figure 2:**
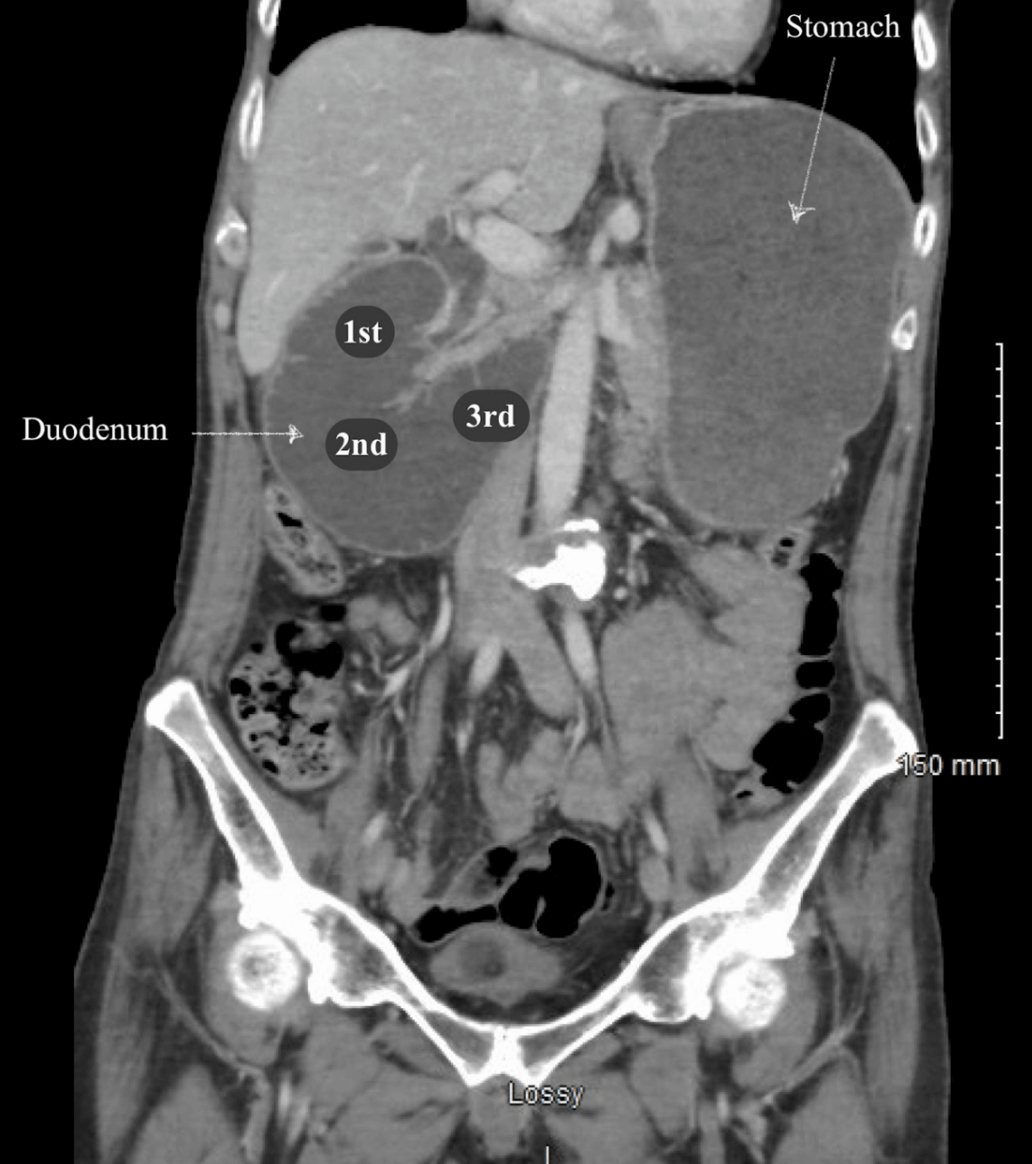
Coronal computed tomography (CT) scan displaying the compression part of the duodenum between the SMA and aorta In this image, the labeled numbers represent the portions of the duodenum. This CT scan highlights compression toward the third portion, indicating characteristic features of superior mesenteric artery (SMA) syndrome. In addition to the compression, the stomach appears to be dilated, possibly due to the obstruction of the gastric outlet.

**Figure 3:**
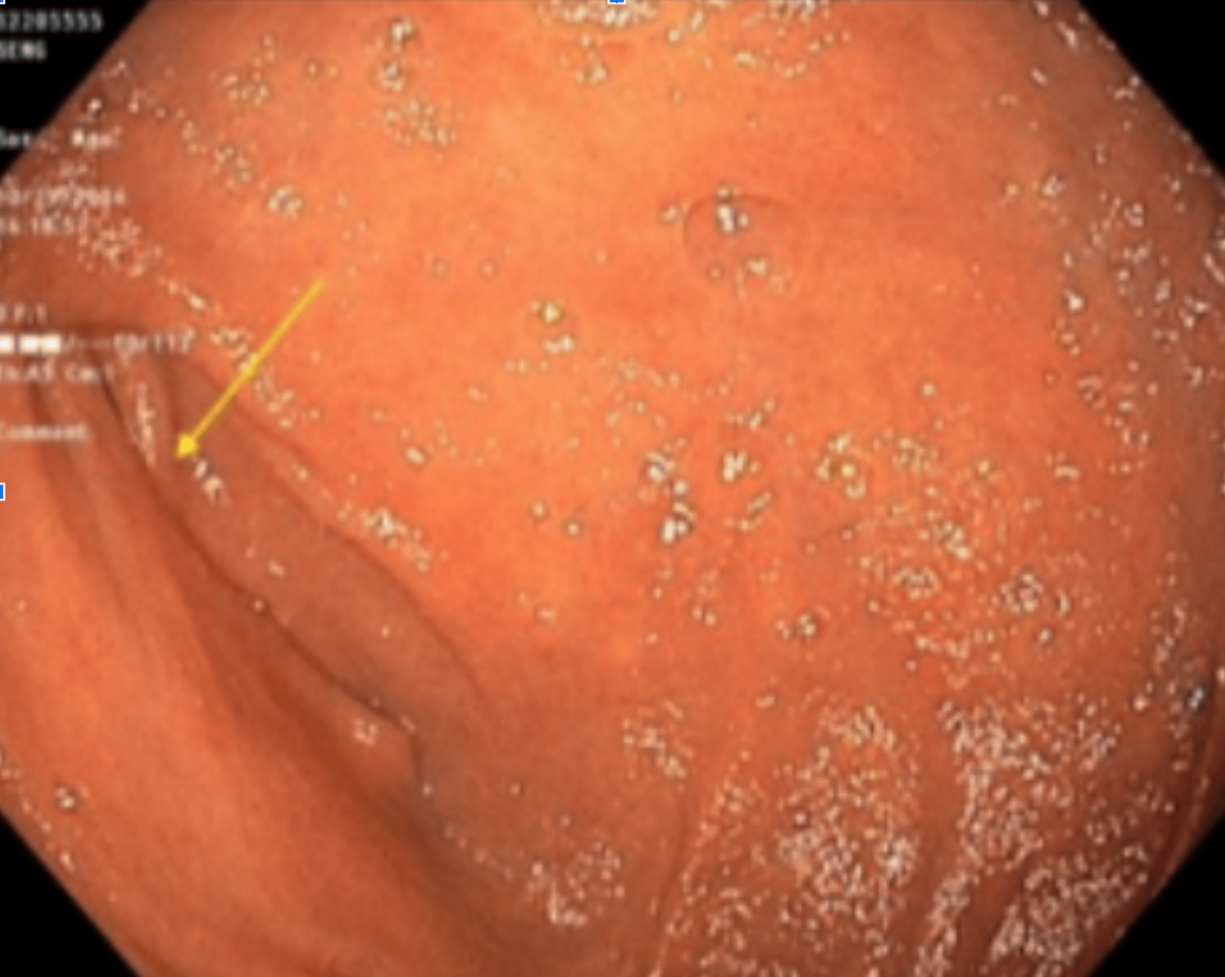
Esophagogastroduodenoscopy of the third portion of the duodenum showing extrinsic compression (yellow arrow)

After stabilization with diazepam, morphine, ondansetron, and two normal saline boluses, axial and coronal CT scans (Figures 1-2) were conducted. The imaging indicated obstruction likely due to adhesions from a prior gastric perforation. An esophagogastroduodenoscopy (EGD) revealed gastritis with compression at the junction of the second and third portions of the duodenum (Figure 3). The stomach was abnormally enlarged, raising suspicion for SMA syndrome or other adhesions. He was supplemented with a jejunostomy Jevity tube to increase the aortomesenteric (AO) angle and to alleviate his symptoms. The laboratory values before discharge (Table 1) were assessed to evaluate the patient’s condition, and previously abnormal results had normalized.

**Table 1:** Values of the laboratory tests conducted nine days after admission K/uL: thousands per cubic milliliter, g/dL: grams per deciliter, mg/dL: milligrams per deciliter, mmol/L: millimoles per liter, U/L: units per liter, BUN: blood urea nitrogen, ALT: alanine transaminase or alanine aminotransferase, AST: aspartate aminotransferase, ALK Phos: alkaline phosphatase, INR: international normalized ratio

Parameters	Result	Reference
White blood count	9.0 K/uL	4.8-10.8 k/uL
Hemoglobin	11.3 g/dL	13.2-17.2 g/dL
Hematocrit	35.0%	41-50%
Platelets	305 mmol/L	150-450 mmol/L
Sodium	137 mmol/L	133-145 mmol/L
Potassium	3.7 mmol/L	3.3-5.1 mmol/L
Chloride	104 mmol/L	96-108 mmol/L
Carbon dioxide	28 mmol/L	22-29 mmol/L
BUN	11 mg/dL	7-20 mg/dL
Creatinine	0.68 mg/dL	0.74-1.35 mg/dL
Anion gap	5 mmol/L	3-10 mmol/L
Calcium	8.8 mg/dL	8.5-10.2 mg/dL
Magnesium	2.3 mg/dL	1.7-2.2 mg/dL
Phosphorus	3.2 mg/dL	2.5-4.5 mg/dL
ALT	53 U/L	7-56 U/L
AST	48 U/L	10-40 U/L
ALK Phos	82 U/L	40-129 U/L
Bilirubin	0.6 mg/dL	0.3-1.2 mg/dL
INR	1.10	0.8-1.1

## Discussion

SMA syndrome is an uncommon disease and a diagnostically challenging condition due to its nonspecific signs and symptoms [4]. The significant risk factors are rapid weight loss and surgical correction of spinal deformities [5]. Based on the current literature, the syndrome is diagnosed through a process of exclusion. They usually recommend a CT to find proximal small bowel obstruction, or an upper GI study can also be used to show delayed contrast passage [2]. Here, in this case, CT was used, but combined with an EGD, Figures 1-2 present notable features, including an abnormal size of the stomach. Furthermore, in Figure 2, the compression of the third portion of the duodenum was apparent. Despite its rarity, actively treating this condition is crucial, as it can lead to significant morbidity and mortality, including malnutrition, dehydration, electrolyte abnormalities, gastric perforation, and gastrointestinal hemorrhage [1]. 

Initial management focuses on conservative measures to address acute symptoms like nausea and vomiting. This includes total parenteral nutrition, electrolyte correction, fluid resuscitation, and gastric decompression through the insertion of a nasogastric tube [1]. It is also important to assess the patient’s recent intake of food and water, as the cycle of symptoms caused by this condition can be detrimental to the patient’s health, with weight loss exacerbating the disease [2]. In addition to acute management, patients are advised to eat small, frequent meals and participate in postural therapies, such as lying in the left lateral decubitus position. Most importantly, nutritional support, such as Jevity in this case, is strongly recommended to increase the mesenteric fat pad and subsequently widen the aortomesenteric (AO) angle. 

If symptoms persist, surgical intervention is recommended, including procedures such as gastrojejunostomy, transabdominal duodenojejunostomy, or laparoscopic duodenojejunostomy [6]. Notably, because the symptoms are nonspecific and no sensitive diagnostic test exists, diagnosis is often delayed, leading to a more severe prognosis. While some patients respond to conservative therapy, most ultimately require surgery [1].

## Conclusions

We presented a case of a one-day history of nausea, vomiting, and abdominal pain. As these symptoms were nonspecific, further tests were performed, including a CT scan and an EGD to confirm the diagnosis. Few patients are alleviated by conservative treatment due to delayed diagnosis. Luckily, in this case, the patient was able to be treated with Jevity without any surgical intervention, restoring his AO angle and reducing his episodes and symptoms related to the syndrome. He was counseled to eat small meals while partaking of postural therapies at discharge to stabilize his condition.

## References

[1] Van Horne N, Jackson JP (2025). Superior mesenteric artery syndrome. StatPearls [Internet].

[2] Scovell S, Hamdan A (2025). Superior mesenteric artery syndrome. UpToDate.

[3] Rabie ME, Ogunbiyi O, Al Qahtani AS, Taha SB, El Hadad A, El Hakeem I (2015). Superior mesenteric artery syndrome: clinical and radiological considerations. Surg Res Pract.

[4] Soqia J, Janoud O, Soukia A, Saadoun R, Mousa K (2024). Challenges and pitfalls in diagnosing superior mesenteric artery syndrome: a case report. SAGE Open Med Case Rep.

[5] Le Moigne F, Lamboley JL, Vitry T (2010). Superior mesenteric artery syndrome: a rare etiology of upper intestinal obstruction in adults. Gastroenterol Clin Biol.

[6] Kingham TP, Shen R, Ren C (2004). Laparoscopic treatment of superior mesenteric artery syndrome. JSLS.

